# What a difference a day makes—female behaviour is less predictable near ovulation

**DOI:** 10.1098/rsos.160998

**Published:** 2017-04-12

**Authors:** Niklas Kästner, S. Helene Richter, Matthias Gamer, Sylvia Kaiser, Norbert Sachser

**Affiliations:** 1 Department of Behavioural Biology, University of Münster, Münster, Germany; 2 Münster Graduate School of Evolution, University of Münster, Münster, Germany; 3 Experimental Clinical Psychology, University of Würzburg, Würzburg, Germany

**Keywords:** animal personality, mice, females, reproductive cycle, anxiety-like behaviour, social interest

## Abstract

‘Animal personalities’ have been shown to exist in many species. Yet, fluctuations in the stability of these inter-individual behavioural differences are not well understood. Against this background, we wondered whether behavioural consistency over time is affected by the reproductive cycle. Female mice were tested twice at an interval of eight weeks in four paradigms assessing social interest as well as anxiety-like behaviour and exploratory locomotion. Twenty-two individuals were tested repeatedly near ovulation, whereas another twenty-two were tested repeatedly in the non-receptive phase. While we found no major behavioural effects at the group level, the reproductive state indeed had profound effects on behavioural stability over time: social interest as well as anxiety-like behaviour proved to be significantly less predictable near ovulation. It is generally believed that phenotypic plasticity is limited due to the costs it brings about. In this context, our data indicate that females accept higher costs in phases directly related to fitness maximization.

## 1. Introduction

Individuality in non-human animals is a fascinating topic that is likewise appealing to both society and science. In fact, everyone who has owned a pet or worked with animals knows that individuals can differ profoundly in their temperament. Over the last few years, this aspect has also gained increasing interest in the scientific community: characterized by inter-individual differences being consistent over time and/or context [1–3], ‘animal personalities’ so far have been described in a wide range of species, including mammals, birds, fish and even invertebrates [4]. Consistency of behavioural differences over time therefore does not mean that individual behaviour is completely inflexible. Rather it means that, in a group of individuals, those being, for example, relatively aggressive/docile at time point 1 are also relatively aggressive/docile at time point 2, irrespective of a change in the total level of aggression that might be displayed by the group. However, while we have learned well enough that stable animal personalities do exist, our knowledge about fluctuations in the stability of such traits is rather limited. Is a personality trait unchangingly stable over time or do certain conditions or states favour higher flexibility? A comprehensive understanding of this phenomenon is of crucial importance not only for behavioural ecology but also for biomedical research as well as for the study of human–animal interactions and animal welfare.

Quite conspicuous changes of state in female mammals occur during the reproductive cycle. Accompanied by fundamental changes in the concentrations of different hormones, it involves a constant transition between two phases: being receptive and not receptive [5]. On a theoretical basis, differences in state have been brought up as an explanation for the coexistence of various personality types in a population [6,7]. Beyond that question, we wondered: can differences in state also result in differences in the stability of personality traits itself, i.e. does the reproductive state affect the degree of behavioural consistency? To investigate this, female mice were tested repeatedly during different reproductive states in established behavioural paradigms assessing common measures to characterize personality types. It has been proposed that phenotypic plasticity is limited due to the costs it brings about [8,9]. For example, to adapt behaviour to a certain environment, information about this environment has to be gathered [8]. As the receptive phase is directly linked to fitness maximization, we expected females to accept higher costs during this phase and thus predicted their behaviour to be less stable near ovulation.

## 2. Animals, material and methods

### 2.1. Animals and housing conditions

This study included 44 female mice of the inbred strain C57BL/6 J purchased from a professional breeder (Charles River Laboratories, Research Models and Services, Germany GmbH, Sulzfeld, Germany) at the age of 21 days. The experiment started with the individuals reaching an age of four months, as in mice of that age the median oestrous cycle length has been shown to be shortest and most stable (less than 5 days) [10]. Throughout the experiment, animals were housed in pairs in polycarbonate cages type III (37 × 21 × 15 cm) with wood shavings as bedding material (Allspan, Höveler GmbH & Co. KG, Langenfeld, Germany), enriched with a semi-transparent red plastic house (11.1 × 11.1 × 5.5 cm, Tecniplast Deutschland GmbH, Hohenpeißenberg, Germany), a wooden stick (approx. 10 × 1.8 × 1.8 cm) and tissue paper as nesting material. Standard mouse diet (Altromin 1324, Altromin GmbH, Lage, Germany) and tap water were available ad libitum. The colony room was maintained at a temperature of about 22°C, a relative humidity of about 50% and with a 12 L: 12 D cycle (lights on at 01.00). Before the experiments, all animals were marked with ear cuts to allow for individual identification. Cages were cleaned and new tissue paper was provided on a weekly basis, whereas the plastic houses and wooden sticks were renewed on a fortnightly basis. In addition to the test animals, eight male and eight female C57BL/6 J mice derived from the internal stock of the Department of Behavioural Biology were used as stimulus animals for the social interest tests.

### 2.2. Experimental design

**Figure 1.**
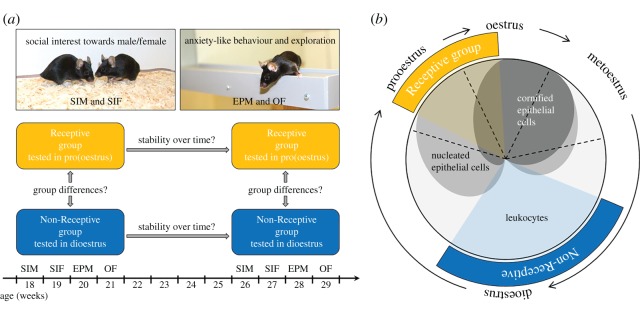
Experimental design and determination of reproductive state. (*a*) To investigate whether behavioural stability over time is affected by the reproductive state, 44 female mice were tested twice at an interval of eight weeks in two social interest tests as well as two tests measuring anxiety-like behaviour and exploratory locomotion. While 22 animals were tested around ovulation (Receptive group), 22 animals were tested in the non-receptive phase (Non-Receptive group). SIM, social interest test with a male stimulus animal; SIF, social interest test with a female stimulus animal; EPM, elevated plus maze; OF, open field test (photos: Dirk-Heinz Loddenkemper). (*b*) On each test day, vaginal smears were examined microscopically. Mice of the Receptive group were tested if no or only a negligible number of leucocytes was present, while nucleated and cornified epithelial cells were present (i.e. in pro-oestrus or oestrus). Mice of the Non-Receptive group were tested when predominantly leucocytes were present (i.e. in dioestrus). (Adapted from Byers *et al*. [11]).

To test for behavioural stability over time, all animals were tested twice at an interval of eight weeks in four behavioural paradigms (figure 1*a*): a social interest test with a male stimulus animal (experimental week, EW, 1 and 9) and with a female stimulus animal (EW 2 and 10), as well as the elevated plus maze (EW 3 and 11) and open field test (EW 4 and 12) measuring anxiety-like behaviour and exploratory locomotion [12].

Prior to the start of the experiment, individuals were pseudo-randomly allocated to one of two experimental groups: the *Receptive* group (*n* = 22, all behavioural tests performed during pro-oestrus/oestrus) and the *Non-Receptive* group (*n* = 22, all behavioural tests performed during dioestrus; figure 1*b*). To balance cage effects, the two individuals housed together never belonged to the same experimental group. Thus, to avoid any bias, it was determined before the beginning of the experiments which animal would perform the experiments during pro-oestrus/oestrus and which during dioestrus. The sample size of *n* = 22 was chosen similar to previous studies investigating stability over time [3,13].

Reproductive states of all animals were determined on a daily basis (see §2.3) and individuals were tested when in the correct state once during each EW. Animals that had not been in the correct state during the EW were treated accordingly, but data were not included in the analysis.

### 2.3. Determination of reproductive state

To assess reproductive states, vaginal smears were performed on each test day between 09.00 and 11.30. For this purpose, the respective mouse was taken out of its cage and put on the metal lid. While holding it by its tail, a small plastic inoculation loop was used to gently sweep over the interior of the vagina. To habituate the mice to this procedure, vaginal smears were already performed on 5 days within the two weeks prior to each test phase.

The smears were dispensed in water and examined microscopically. The decision was made by an experienced experimenter (N.K.) based on the cytology as described by other authors [5,10,11]. Mice of the Receptive group were only tested on a day when they were assessed to be in progressing pro-oestrus or in oestrus, mice of the Non-Receptive group when they were assessed to be in dioestrus. To avoid an effect of the vaginal smears on the test performance, at least 1.5 h passed between determination of reproductive state and testing. To ensure that during testing the mice were still in the correct state, those individuals assessed to be already in late dioestrus or oestrus, respectively, were not tested (figure 1*b*).

### 2.4. Behavioural testing

All behavioural tests were performed during the dark period between 13.00 and 17.00, i.e. the animals’ active phase, by an experienced experimenter (N.K.). Individual testing order was pseudo-randomized on each test day and balanced between the experimental groups. All equipment used for the tests was cleaned with 70% ethanol between subjects.

The social interest tests were performed in the housing room under red light conditions and behaviour was recorded by live observation. Elevated plus maze and open field test were performed in a room separate from the subjects’ housing room. Behaviour was recorded by a camera (Logitech Webcam Pro 9000) and the animals’ movements were automatically analysed by the video-tracking system ANY-maze (v. 4.75, Stoelting Co., Wood Dale, USA). Varying sample sizes result from the fact that some individuals could not be tested in the correct oestrous state. The experimenter was not blind to the reproductive state of the individuals. This was not considered to confound the results, as nine out of 11 parameters were assessed and analysed automatically by a computer software.

#### 2.4.1. Social interest tests

The social interest tests were a modified version of a test described in [14]. Subjects were taken out of the home cage and placed in the test arena that consisted of a standard polycarbonate type III cage (37 × 21 × 15 cm) containing a thin layer of wood shavings. A transparent plastic cover was placed on the arena to prevent the mice from jumping out. After a habituation phase of 1 min, a cylindric wire mesh cage (diameter: 10 cm, height: 8 cm) was put in the middle of the arena and the mice could freely explore the set-up for 3 min to become accustomed to it. For the actual test, a stimulus animal was then introduced into the wire mesh cage: either an unfamiliar male mouse (social interest test male, SIM) or an unfamiliar female mouse not in oestrus (social interest test female, SIF). Now, subjects could explore the arena for another 3 min and the behaviour was recorded. The measure for social interest was the time the focal mouse investigated (i.e. was sniffing at) the cage containing the stimulus animal.

#### 2.4.2. Elevated plus maze

The apparatus consisted of a plus-shaped maze that was elevated 50 cm above the floor. The maze comprised four arms (30 × 5 cm each) as well as a central square (5 × 5 cm). Two opposite arms were surrounded by 20 cm high wooden walls (closed arms), the two remaining arms were enclosed only by a 0.4 cm high border to prevent the mice from falling from the maze (open arms). The apparatus was made of wood painted light grey and the surface of the maze was covered by a grey PVC inlay. The illumination level in the centre square was set to 25 lux.

Subjects were transported to the testing room in an empty polycarbonate type II cage. After 1 min in the empty cage, individuals were placed in the centre square of the elevated plus maze (EPM) with the head in the direction of always the same closed arm. Now the experimenter left the room and the apparatus could be explored freely by the mouse for 5 min. Measures taken were relative time on open arms (time on open arms/(time on open + time on closed arms)), relative number of open arm entries (open arm entries/(open arm entries + closed arm entries)), latency to enter an open arm and distance travelled on the open arms (anxiety-like behaviours) as well as the total path travelled (exploratory locomotion).

#### 2.4.3. Open field test

The open field (OF) was completely made of white coated plywood and consisted of a square arena (80 × 80 cm) surrounded by walls (42 cm). The illumination level was set to 25 lux. After having been transported to the testing room in an empty polycarbonate type II cage, mice were placed in a black cylinder (diameter: 11 cm, height: 20 cm) that was located in always the same corner of the arena. After 1 min, the cylinder was lifted, the experimenter left the room and the apparatus could be explored freely by the mouse for 5 min. Measures taken were time in the centre (defined as the area being located at least 20 cm distant from the walls), number of centre entries, distance travelled in the centre (anxiety-like behaviours) as well as the total distance travelled (exploratory locomotion).

### 2.5. Statistical analyses

As data did not comply with the requirements of parametric statistics, non-parametric statistics were applied. Behavioural group-level differences between the Receptive and the Non-Receptive group were analysed using the Mann–Whitney *U*-test (two-tailed). Additionally, to compare the behavioural performance within group between the two test rounds at the group level, the Wilcoxon signed-rank test was applied (two-tailed).

To compare the overall degree of behavioural consistency over time between the Receptive and the Non-Receptive group, we followed a random regression approach [15]. In detail, we fitted a regression model with the data of the second behavioural tests as the dependent variable and the data of the first behavioural tests as the predictor using the software R v. 3.2.3 (R Development Core Team 2015). Additional moderating factors included in the model were ‘group’ (Receptive/Non-Receptive) as well as ‘test’ (= all 11 parameters). A random intercept for each animal was defined to account for the repeated measurement of the same individual. For the computation of the model, values were *z*-standardized within each group and each test. As the data were not normally distributed, a robust regression was applied (R-package robustlmm v. 1.8).

To further illustrate the data, correlations between time points for each parameter within each group were calculated using Spearman's rank correlation. As the concept of stability over time comprises only positive correlations (i.e. the more in the first, the more in the second test phase), the analysis was run one-tailed (e.g. [13]). These statistical tests were conducted using the software package SPSS (IBM, v. 22 for Windows).

Across statistical analyses, differences were considered to be significant at *p* < 0.05.

## 3. Results

At the group level, there was no effect of the reproductive state in 10 out of 11 measured parameters in the first test phase (table 1). The only difference concerned locomotion in the OF, where mice of the Receptive group travelled significantly less (*n*_Receptive_ = 19, *n*_Non-Receptive_ = 20, *U* = 91, *p* = 0.005). In the second test phase, the groups did not differ in any of the 11 measured parameters (table 1). In both groups, in nine of 11 parameters anxiety-like behaviour increased and exploratory locomotion as well as social interest decreased significantly from the first to the second test phase, possibly reflecting habituation to the task (electronic supplementary material, table S3).

**Table 1.** Summary of behavioural performance of mice of the Receptive and the Non-Receptive group in behavioural tests of the first and second test phase. SIM, social interest test with male stimulus animal; SIF, social interest test with female stimulus animal; EPM, elevated plus maze; OF, open field test. Data of both test phases are presented as medians with first (Q1) and third (Q3) quartiles for the Receptive and Non-Receptive group, respectively. Statistics: Mann–Whitney *U*-test, two-tailed; italicized number: *p* < 0.05.

		behavioural tests I						behavioural tests II					
		Receptive		Non-Receptive		statistics		Receptive		Non-Receptive		statistics	
		(Q1; Q3) median	*n*	(Q1; Q3) median	*n*	*U*	*p*	(Q1; Q3) median	*n*	(Q1; Q3) median	*n*	*U*	*p*
SIM	investigating stimulus (s)	107.00 (76.00; 122.00)	21	94.50 (80.50; 118.00)	22	193.5	0.369	52.00 (30.00; 59.00)	17	48.50 (26.00; 51.75)	18	125.0	0.364
SIF	investigating stimulus (s)	53.00 (41.25; 72.75)	20	59.00 (49.00; 75.50)	20	165.0	0.351	61.50 (48.25; 78.75)	18	57.00 (53.00; 73.00)	17	148.0	0.877
EPM	total path travelled (m)	7.02 (6.15; 7.67)	19	8.08 (5.95; 9.18)	21	148.0	0.169	5.11 (3.79; 5.70)	18	4.84 (3.94; 6.54)	19	153.0	0.599
	open arm entries (rel.)	0.32 (0.20; 0.38)	19	0.32 (0.30; 0.40)	21	159.5	0.285	0.08 (0.02; 0.17)	18	0.09 (0.06; 0.18)	19	145.5	0.444
	open arm time (rel.)	0.19 (0.16; 0.29)	19	0.20 (0.16; 0.32)	21	179.0	0.592	0.01 (0.00; 0.04)	18	0.03 (0.01; 0.10)	19	134.0	0.267
	open arm distance (m)	0.95 (0.59; 1.42)	19	0.99 (0.45; 1.88)	21	181.0	0.630	0.01 (0.00; 0.11)	18	0.05 (0.00; 0.47)	19	146.0	0.433
	open arm latency (s)	13.90 (8.60; 19.05)	19	13.00 (9.80; 20.00)	21	196.0	0.931	128.95 (26.70; 299.50)	18	82.20 (12.10; 199.80)	19	140.5	0.359
OF	total path travelled (m)	26.24 (20.87; 31.01)	19	32.24 (27.26; 34.46)	20	91.0	*0.005*	21.27 (18.74; 25.40)	19	23.59 (21.40; 27.88)	21	138.0	0.098
	centre entries (no.)	10.00 (8.50; 13.00)	19	12.00 (8.00; 16.25)	20	142.0	0.180	4.00 (2.00; 6.00)	19	4.00 (3.00; 9.00)	21	165.5	0.361
	centre time (s)	14.90 (12.65; 19.15)	19	21.50 (13.88; 26.38)	20	134.0	0.118	9.50 (5.35; 20.65)	19	11.50 (5.30; 20.30)	21	184.0	0.688
	path centre (m)	2.52 (1.93; 3.26)	19	3.72 (2.07; 4.51)	20	133.0	0.113	0.84 (0.74; 1.59)	19	1.24 (0.85; 2.40)	21	159.0	0.279

**Figure 2.**
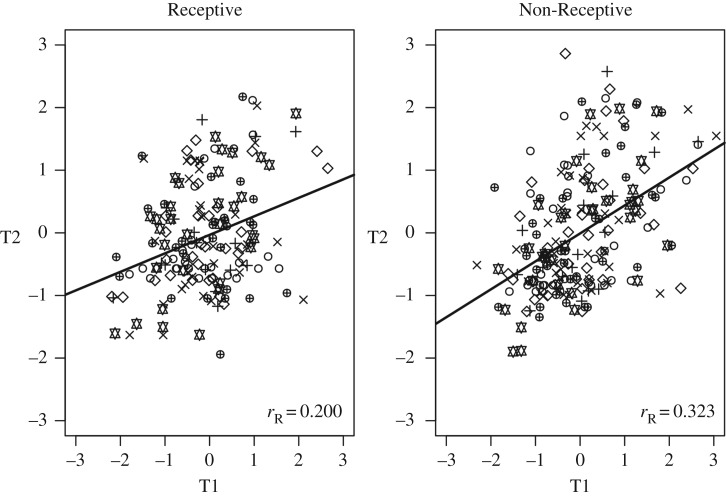
Stability over time across behavioural tests. Behavioural consistency over time across tests was higher in the Non-Receptive group (201 data points from *n* = 22 animals) when compared with the Receptive group (172 data points from *n* = 22 animals), indicated by a significant global interaction (*p* ≤ 0.028) of T1 (first behavioural test) and group (Receptive/Non-Receptive) on T2 (second behavioural test). Statistics: robust regression of T1 (predictor) on T2 (criterion) with group and test (all 11 parameters) as moderating factors and a random intercept for each animal to account for the repeated measurement of the same individual. Different symbols indicate different test parameters. Lines represent robust regressions of T2 on T1 as a function of group. *r*_R_: robust correlation coefficient as calculated by the minimum covariance determinant method [16].

While the reproductive state did not affect behaviour at the group level, it indeed had profound effects on behavioural consistency over time (figure 2). To statistically confirm this difference between the two groups across behavioural tests, a robust regression of the first (T1, predictor) on the second behavioural test (T2, criterion) was conducted with group and test as moderating factors. Indeed, in addition to significant interaction effects between the predictor and specific behavioural tests across groups (OF, total path travelled, *p* = 0.044; SIF, *p* = 0.018, and SIM investigating stimulus time, *p* = 0.014), the model revealed a significant global interaction of the predictor and the group factor on the criterion (*p* = 0.028), indicating an overall higher stability in the Non-Receptive group (figure 2).

Additionally, to further illustrate temporal stability across behavioural tests, a Spearman's rank correlation was calculated for each parameter within each group. In the Non-Receptive group, behaviour was significantly correlated between time points in eight out of 11 measured parameters with high correlation coefficients (*r*_S_ = 0.4–0.7). Conversely, there were no significant correlations in the Receptive group (table 2). More specifically, in the Non-Receptive group, social interest was correlated over time in terms of investigative behaviour towards a male (SIM; *r*_S_ = 0.656, *p* = 0.002, *n* = 18) and a female stimulus animal (SIF; *r*_S_ = 0.729, *p* = 0.001, *n* = 17). Furthermore, anxiety-like behaviour and exploratory locomotion were correlated over time in the EPM concerning total path travelled (*r*_S_ = 0.453, *p* = 0.030, *n* = 18), path travelled on the open arms (*r*_S_ = 0.469, *p* = 0.025, *n* = 18), relative time on open arms (*r*_S_ = 0.415, *p* = 0.043, *n* = 18) and latency to enter the open arms (*r*_S_ = 0.447, *p* = 0.031, *n* = 18) as well as in the OF concerning number of centre entries (*r*_S_ = 0.643, *p* = 0.002, *n* = 19) and path travelled in the centre (*r*_S_ = 0.570, *p* = 0.006, *n* = 19).

**Table 2.** Behavioural stability over time in the Receptive and Non-Receptive group. SIM: social interest test with a male stimulus animal; SIF, social interest test with a female stimulus animal; EPM, elevated plus maze; OF, open field test. Correlations were calculated between performance in the first and the second test phase. The degree of stability over time is indicated by the correlation coefficient *r*_S_. Statistics: Spearman's rank correlation, one-tailed; italicized numbers: *p* < 0.05.

		Receptive			Non-Receptive		
		*r*_S_	*p*	*n*	*r*_S_	*p*	*n*
SIM	investigating stimulus (s)	0.356	0.088	16	*0*.*656*	*0.002*	18
SIF	investigating stimulus (s)	0.397	0.058	17	*0.729*	*0.001*	17
EPM	total path travelled (m)	−0.275	0.161	15	*0.453*	*0.030*	18
	open arm entries (rel.)	0.370	0.088	15	0.338	0.085	18
	open arm time (rel.)	0.220	0.216	15	*0.415*	*0.043*	18
	open arm distance (m)	0.299	0.140	15	*0.469*	*0.025*	18
	open arm latency (s)	−0.351	0.100	15	*0.447*	*0.031*	18
OF	total path travelled (m)	0.405	0.060	16	0.225	0.178	19
	centre entries (no.)	0.328	0.108	16	*0.643*	*0.001*	19
	centre time (s)	0.147	0.293	16	0.246	0.155	19
	path centre (m)	0.129	0.316	16	*0.570*	*0.005*	19

## 4. Discussion

Here, we show that the difference in a single state—Receptive/Non-Receptive—can lead to a profound difference in behavioural consistency over time: in a group of female mice of the same age and with identical genetic make-up, behaviour was less predictable near ovulation. The term ‘behavioural consistency’ refers to the fact that behaviour is stable *relative to the behaviour of other individuals*—irrespective of whether general behavioural characteristics at the group level are susceptible to temporal changes. In this study, values generally decreased significantly from the first to the second test, which could reflect habituation to the task or other unspecific effects. Yet, this does not conflict with the concept of ‘behavioural consistency’ as depicted above: inter-individual differences were considerably stable over time—and this was significantly more pronounced in the Non-Receptive group.

At first glance, these results might seem surprising. However, they correspond to theoretical work on the question why evolution has favoured personalities instead of maximal behavioural plasticity. In fact, it is not trivial to explain why an individual does not adapt its behaviour freely to each situation [1]. It has been suggested that phenotypic plasticity is limited because it creates a number of costs, among them the costs of collecting correct information about the environment [1,8,9]. These could include energetic costs or risks of predator inspection [8]. Owing to these costs, it might pay off to have stable behavioural traits instead of investing in high plasticity [9]. As in our study behaviour was less predictable during the receptive phase, we conclude that higher costs are accepted during states directly linked to fitness maximization.

Up to now, there is some evidence that environmental changes can affect the stability of behaviour [17]. Additionally, it has been suggested that fluctuations in the consistency of behavioural traits and stress responsiveness may occur during sensitive phases of life [13,18]. Our data now demonstrate that, even in fully adult individuals living in a non-fluctuating environment, the degree of behavioural consistency over time can undergo pronounced changes within a few days.

Concerning underlying mechanisms, most promising candidates are the reproductive hormones. Concentrations of oestrogen and progesterone vary predictively along the reproductive cycle [5,19]. They not only trigger the related physiological processes, but also exert behavioural effects [19,20], and can significantly impact mood [21]. It could well be possible that these hormones also control the degree of behavioural stability. This hypothesis, however, has not been tested in any species, yet.

From a biomedical perspective, this study addresses another highly topical question: the female oestrous cycle is the reason for an enormous male bias in neuroscience and biomedical rodent research [22]. Among other things, differences in reproductive state are believed to decrease the homogeneity of study populations and confound effects of experimental manipulations. In line with other studies [23,24] and a recent meta-analysis [25], our data, however, revealed no major effect of the reproductive state on the measured behaviours at the group level.

To conclude, we show for the first time that the temporal stability of animal personality traits can change within a few days depending on the state, with behaviour being less predictable in the receptive phase. Future comparable studies in other species, including research in evolutionary psychology, might prove this as a general principle.

## Supplementary Material

Raw data

## Supplementary Material

Results of the Wilcoxon signed-rank test

## Data accessibility

The data that support the findings of this study are provided as the electronic supplementary material.
