# Supplementary material for: What a difference a day makes—female behaviour is less predictable near ovulation
Source: R Soc Open Sci. 2017 Apr 12;4(4):160998. doi: 10.1098/rsos.160998 (PMC5414256; doi:10.1098/rsos.160998)
Supplement: Results of the Wilcoxon signed-rank test [file rsos160998supp2.docx]

**Table 3: Summary of behavioural performance of mice of the Receptive and the Non-Receptive group in behavioural tests of the first and second test phase – comparison between the test rounds.**

|  |  | **Receptive** | | | | | | **Non-Receptive** | | | | |
| --- | --- | --- | --- | --- | --- | --- | --- | --- | --- | --- | --- | --- |
|  |  |  |  |  |  |  |  |  |  |  |  |  |
|  |  | **Behavioural**  **tests I** |  | **Behavioural**  **tests II** | **Statistics** | | | **Behavioural**  **tests I** | **Behavioural**  **tests II** | **Statistics** | | |
|  |  |  |  |  |  |  |  |  |  |  |  |  |
|  |  | **Median** |  | **Median** | **n** | **Z** | **p** | **Median** | **Median** | **n** | **Z** | **p** |
|  |  | **(Q1; Q3)** |  | **(Q1; Q3)** |  |  |  | **(Q1; Q3)** | **(Q1; Q3)** |  |  |  |
| **SIM** | investigating stimulus (s) | 106.00 |  | 54.00 | 16 | -3.517 | **0.000** | 94.50 | 48.50 | 18 | -3.724 | **0.000** |
|  |  | (70.50; 116.00) |  | (42.00; 59.00) |  |  |  | (76.75; 124.00) | (26.00; 52.00) |  |  |  |
| **SIF** | investigating stimulus (s) | 53.00 |  | 62.00 | 17 | -1.374 | 0.178 | 59.00 | 57.00 | 17 | -0.181 | 0.870 |
|  |  | (39.00; 63.00) |  | (48.00; 69.00) |  |  |  | (49.00; 78.00) | (53.00; 73.00) |  |  |  |
| **EPM** | total path travelled (m) | 7.02 |  | 5.11 | 15 | -2.953 | **0.002** | 7.22 | 5.22 | 18 | -3.245 | **0.000** |
|  |  | (5.90; 7.35) |  | (3.95; 5.51) |  |  |  | (5.87; 9.34) | (4.01; 6.69) |  |  |  |
|  | open arm entries (rel.) | 0.32 |  | 0.08 | 15 | -3.351 | **0.000** | 0.32 | 0.09 | 18 | -3,724 | **0.000** |
|  |  | (0.24; 0.38) |  | (0.03; 0.11) |  |  |  | (0.26; 0.40) | (0.05; 0.18) |  |  |  |
|  | open arm time (rel.) | 0.18 |  | 0.01 | 15 | -3.408 | **0.000** | 0.20 | 0.03 | 18 | -3.636 | **0.000** |
|  |  | (0.16; 0.25) |  | (0.00; 0.02) |  |  |  | (0.13; 0.29) | (0.01; 0.10) |  |  |  |
|  | open arm distance (m) | 0.95 |  | 0.00 | 15 | -3.408 | **0.000** | 1.00 | 0.11 | 18 | -3.549 | **0.000** |
|  |  | (0.59; 1.17) |  | (0.00; 0.09) |  |  |  | (0.43; 1.88) | (0.00; 0.47) |  |  |  |
|  | open arm latency (s) | 16.90 |  | 186.10 | 15 | -2.897 | **0.002** | 13.65 | 93.45 | 18 | -3.070 | **0.001** |
|  |  | (9.30; 18.68) |  | (41.85; 298.50) |  |  |  | (10.13; 20.10) | (16.78; 233.00) |  |  |  |
| **OF** | total path travelled (m) | 25.42 |  | 21.16 | 16 | -2.068 | **0.039** | 32.14 | 23.59 | 19 | -3.058 | **0.001** |
|  |  | (19.71; 30.25) |  | (19.03; 25.35) |  |  |  | (27.04; 34.17) | (21.89; 28.54) |  |  |  |
|  | centre entries (no.) | 9.50 |  | 4.50 | 16 | -3.302 | **0.000** | 13.00 | 5.00 | 19 | -3.619 | **0.000** |
|  |  | (7.50; 11.50) |  | (2.00; 6.00) |  |  |  | (9.00; 16.00) | (3.00; 9.75) |  |  |  |
|  | centre time (s) | 14.40 |  | 9.90 | 16 | -1.241 | 0.231 | 21.50 | 11.50 | 19 | -1.690 | 0.094 |
|  |  | (12.83; 18.10) |  | (5.58; 28.98) |  |  |  | (15.75; 26.93) | (5.00; 19.65) |  |  |  |
|  | path centre (m) | 2.48 |  | 0.99 | 16 | -2.999 | **0.001** | 3.77 | 1.24 | 19 | -4.015 | **0.000** |
|  |  | (1.63; 3.22) |  | (0.75; 1.36) |  |  |  | (2.47; 4.32) | (0.83; 2.59) |  |  |  |

SIM: social interest test with male stimulus animal, SIF: social interest test with female stimulus animal, EPM: Elevated Plus Maze, OF: Open Field Test. Data of both test phases are presented as medians with 1^st^ (Q1) and 3^rd^ (Q3) quartiles for the Receptive and Non-Receptive group, respectively. Statistics: Wilcoxon signed-rank test, 2-tailed; bold numbers: p < 0.05. Please note: n-values and medians differ from those in table 1, as the Wilcoxon signed-rank test only considers individuals that had been tested during both test phases.
